# 

**Published:** 1933

**Authors:** 


### FOREWORD

For the compilation of this Index we are indebted to the Assistant Editor (Mr. E. Watson-Williams) and the Senior Assistant in the University Library (Mr. A. E. S. Roberts).

Throughout it references are given to volume and page; the year of publication of each volume can be found from the list on pages 5–8. The Index to Authors gives the authors' names in alphabetical order, followed by the titles of the contributions of each in *chronological* order. The Index to Subjects is not exactly a "title" index, as owing to the variety of titles sometimes chosen for a single subject this is probably the most useful arrangement. As far as possible all papers dealing with one subject are collected under a single heading: where this has proved impracticable, a list of relevant titles follows the principal subject-heading, *e.g.* under Eye, History. Only one entry, as a rule, is given for each paper, but cross-references are given for all important nouns and adjectives occurring in titles, and the exact title of any paper can be found by referring to the appropriate entry in the Index to Authors.

In general, diseases are listed in preference to organs—*Cancer* of breast, pulmonary *tuberculosis*: but convenience in reference, rather than any strictly logical arrangement, has been the deciding factor. Where two or more synonyms exist, the most frequently used has been chosen.

Only original articles, case reports and editorial and obituary notices have been indexed; lack of space excluding meetings of societies, local medical notes, comments, abstracts, "scraps," reviews, etc.

### EDITORIAL STAFF

From 1883 to the formation of the Committee in 1891 the publication of the *Journal* was entirely the work of J. Greig Smith and L. M. Griffiths.

**Editor.**

| | |
|---|---|
| J. Greig Smith .. .. .. .. | 1883–1892 |
| R. Shingleton Smith .. .. .. | 1892–1912 |
| P. Watson-Williams .. .. .. | 1912–1926 |
| [1]J. A. Nixon .. .. .. .. | 1926 |

**Assistant Editor.**

| | |
|---|---|
| [2]L. M. Griffiths .. .. .. .. | 1883–1899 |
| P. Watson-Williams .. .. .. | 1900–1912 |
| J. A. Nixon .. .. .. .. | 1912–1926 |
| Carey F. Coombs .. .. .. .. | 1926–1932 |
| [1]E. Watson-Williams .. .. .. | 1933 |

**Editorial Secretary.**

| | |
|---|---|
| B. M. H. Rogers .. .. .. .. | 1895–1898 |
| J. Taylor .. .. .. .. .. | 1899–1909 |
| J. A. Nixon .. .. .. .. | 1909–1912 |
| J. M. Fortescue-Brickdale .. .. | 1913–1921 |
| [3]W. A. Smith .. .. .. .. | 1915–1918 |
| [1]A. L. Flemming .. .. .. .. | 1921 |

**Treasurer of the Medico-Chirurgical Society, ex-officio.**

| | |
|---|---|
| [1]A. E. Iles .. .. .. .. .. | 1926 |

**Committee.**

| | |
|---|---|
| Barclay J. Baron .. .. .. | 1891–1893 |
| J. Michell Clarke .. .. .. | 1891–1918 |
| F. R. Cross .. .. .. .. .. | 1891–1898 |
| W. H. Harsant .. .. .. .. | 1891–1899 |
| J. Swain .. .. .. .. .. | 1897–1924 |
| B. M. H. Rogers .. .. .. .. | 1898–1900 |
| J. Lacy Firth .. .. .. .. | 1900–1926 |
| Carey F. Coombs .. .. .. .. | 1919–1926 |
| [1]E. W. Hey Groves .. .. .. | 1924 |
| E. Watson-Williams .. .. .. | 1926–1933 |
| [1]A. Rendle Short .. .. .. .. | 1933 |

[1] Present Committee.

[2] And Publications Secretary, 1883–1895.

[3] During absence of Captain Fortescue-Brickdale on service.

### PRESIDENTS OF THE MEDICO-CHIRURGICAL SOCIETY AND PRESIDENTIAL ADDRESSES

| Vol. | Year. | President | Address |
|---|---|---|---|
| | 1874. | F. Brittan. | |
| | 1875. | R. W. Coe. | |
| | 1876. | S. Martyn. | |
| | 1877. | A. Prichard. | |
| | 1878. | H. E. Fripp. | |
| | 1879. | W. Michell Clarke. | |
| | 1880. | J. G. Swayne. | |
| | 1881. | E. Long Fox. | |
| | 1882. | J. G. Davey. | |
| **1.** | 1883. | W. J. Fyffe .. | Malarial Poisoning, **1,** 143. |
| **2.** | 1884. | G. F. Burder .. | Etiology of Catarrh, **2,** 217. |
| **3.** | 1885. | W. H. Spencer .. | Pyrexia, **3,** 217. |
| **4.** | 1886. | F. P. Lansdown | Progress of Practical Surgery, **4,** 217. |
| **5.** | 1887. | L. M. Griffiths .. | Shakespeare and the Medical Sciences, **5,** 225. |
| **6.** | 1888. | R. Shingleton Smith. | Phthisis and the Germ Theory, **6,** 225. |
| **7.** | 1889. | N. C. Dobson .. | Surgical Aspects of Carcinoma, **7,** 225. |
| **8.** | 1890. | S. H. Swayne. | |
| **9.** | 1891. | F. R. Cross .. .. | Medical Progress, **9,** 241. |
| **10.** | 1892. | E. M. Skerritt .. | The Teachings of Failure, **10,** 229. |
| **11.** | 1893. | J. Greig Smith .. | Modern Medical Journalism, **11,** 217. |
| **12.** | 1894. | A. J. Harrison .. | Comparative Pathology, **12,** 273. |
| **13.** | 1895. | A. W. Prichard | School Surgery, **13,** 241. |
| **14.** | 1896. | A. E. Aust Lawrence. | The Evils of Marriage and Pregnancy in Women who do not possess Sound Pelvic Organs, **14,** 289. |

| | | | |
|---|---|---|---|
| **15.** | 1897. | J. E. SHAW .. .. | Some Points in Lunacy Practice, **15,** 301. |
| **16.** | 1898. | R. ROXBURGH .. | Progress and Practice, **16,** 285. |
| **17.** | 1899. | W. H. HARSANT .. | Medical Bristol in the Eighteenth Century, **17,** 297. |
| **18.** | 1900. | D. S. DAVIES .. | Modern Aspects of Preventive Medicine, **18,** 289. |
| **19.** | 1901. | B. J. BARON .. | Progress in Treatment of Throat, Nose and Ear Disease, **19,** 289. |
| **20.** | 1902. | G. MUNRO SMITH | The Medical Life, **20,** 289. |
| **21.** | 1903. | J. P. BUSH .. .. | Some Surprises in my Professional Career, **21,** 289. |
| **22.** | 1904. | R. EAGER .. .. | Problems in Psychology, **22,** 289. |
| **23.** | 1905. | J. DACRE .. .. | The Family Doctor, **23,** 289. |
| **24.** | 1906. | J. TAYLOR .. .. | X-ray Therapeutics, **24,** 289. |
| **25.** | 1907. | H. WALDO .. .. | Syphilis, **25,** 289. |
| **26.** | 1908. | J. M. CLARKE .. | Leukæmia and Some Allied Affections, **26,** 289. |
| **27.** | 1909. | J. SWAIN .. .. | History and Practice of Surgery, **27,** 289. |
| **28.** | 1910. | B. M. H. ROGERS | The Medical Aspect of Boswell's *Life of Johnson*, **28,** 289. |
| **29.** | 1911. | C. A. MORTON .. | Present Position of Surgery of Malignant Growths, **29,** 289. |
| **30.** | 1912. | W. C. SWAYNE .. | The Problem of Medical Education, **30,** 316. |
| **31.** | 1913. | P. WATSON-WILLIAMS. | Specialization and the Medical Curriculum, **31,** 289. |

| | | | |
|---|---|---|---|
| 32. | 1914. | W. H. C. NEWNHAM | Evolution of Gynæcology, **32,** 257. |
| 33. | 1915. | G. PARKER .. .. | The Foundation of the Bristol Medico-Chirurgical Society, **33,** 129. |
| 34. | 1916. | F. H. EDGEWORTH | The Art of Medicine in the Age of Homer, **34,** 105. |
| 35. | 1917. | F. LACE .. .. | Some Developments in Abdominal Surgery, **36,** 1. |
| 36. | 1918. | R. G. P. LANSDOWN | The Systematic Examination of the Abdomen, **36,** 33. |
| | 1919. | I. W. HALL .. .. | Contemporary Medicine from the Standpoint of Pathology, **36,** 105. |
| 37. | 1920. | L. E. V. EVERY-CLAYTON. | Medicine and Surgery, **37,** 193. |
| 38. | 1921. | C. H. WALKER .. | Quackery in Relation to Diseases of the Eye, **38,** 129. |
| 39. | 1922. | J. A. NIXON .. .. | The Debt of Medicine to the Fine Arts, **40,** 1. |
| 40. | 1923. | J. L. FIRTH .. .. | Evolutional History of Renal Surgery and of Temporal Bone Surgery, **41,** 1. |
| 41. | 1924. | J. O. SYMES .. | Postgraduate Medical Education, **42,** 1. |
| 42. | 1925. | T. CARWARDINE .. | Continuity and Change, **42,** 201. |
| 43. | 1926. | A. L. FLEMMING .. | Anæsthesia, **44,** 1. |
| 44. | 1927. | W. K. WILLS .. | Some Developments in Dermatology during the last Thirty Years, **45,** 1. |
| 45. | 1928. | H. L. ORMEROD .. | The Care and Treatment of Mental Defectives, **46,** 1. |

| | | | |
|---|---|---|---|
| **46.** | 1929. | C. F. WALTERS .. | The Duty of Medicine to the Race, **47,** 1. |
| **47.** | 1930. | D. C. RAYNER .. | The Relation of Gynæcology to the Glands of Internal Secretion, **48,** 1. |
| **48.** | 1931. | J. R. CHARLES .. | Relationship between Structure and Function, **49,** 1. |
| **49.** | 1932. | E. W. H. GROVES | A Surgical Adventure (autobiography), **50,** 1. |
| **50.** | 1933. | H. E. HARRIS .. | Looking Back (**51,** 1). |

### THE LONG FOX MEMORIAL LECTURES

| | | | |
|---|---|---|---|
| I. | 1904. | J. Beddoe .. | General and Biographical, **22,** 303. |
| II. | 1905. | R. S. Smith .. | Graves's Disease, **23,** 317. |
| III. | 1906. | A. J. Harrison | Dermatitis from without and Dermatitis from within, **24,** 325. |
| IV. | *1907. | P. Watson-Williams. | Suppurative Disease in Nose and Ear, **26,** 1. |
| V. | 1908. | J. H. Parsons .. | Metastatic Inflammations of the Eye, **27,** 1. |
| VI. | 1909. | E. Fawcett .. | Development of the Human Skull, **28,** 97. |
| VII. | 1910. | J. M. Clarke .. | Cerebro-spinal Syphilis, **29,** 1. |
| VIII. | 1913. | F. H. Edgeworth | The Cranial Nerves, their Motor Nuclei and Muscle Sensory Fibres. |
| IX. | 1915. | F. R. Cross .. | Evolution of the Sense of Sight, **33,** 163. |
| X. | 1918. | A. F. S. Kent .. | Industrial Fatigue. |
| XI. | 1921. | E. W. H. Groves | The Repair of Bone Injuries, **38,** 142. |
| XII. | 1923. | F. Francis .. | Relation between Chemistry and Medicine, **40,** 119. |
| XIII. | 1924. | C. Lloyd Morgan | The Conditioned Response, **41,** 166. |
| XIV. | 1925. | Carey F. Coombs | Aetiology of Cardiac Disease, **43,** 1. |
| XV. | 1926. | G. A. Buckmaster | The Cells and Liquids of the Body, **44,** 154. |

* In 1907 the Committee decided that the *Bristol Medico-Chirurgical Journal* should have the option of publishing the Lecture.

| | | | |
|---|---|---|---|
| XVI. | 1927. | H. DEVINE .. | The Reality of Delusions, **45,** 19. |
| XVII. | 1928. | J. O. SYMES .. | The Relationship of Erythema Nodosum to Tuberculosis, **45,** 233. |
| XVIII. | 1929. | A. R. SHORT .. | Ten Years' Progress in Surgical Treatment, **46,** 243. |
| XIX. | 1930. | J. A. NIXON .. | Influence of Food on the Production and Prevention of Disease, **47,** 255. |
| XX. | 1931. | A. L. FLEMMING | The Partnership between Anæsthesia and Surgery, **48,** 233. |
| XXI. | 1932. | A. J. M. WRIGHT | The Problem of Deafness, **49,** 256. |
| XXII. | 1933. | C. A. JOLL .. | Recent Advances in Diagnosis and Treatment of Cancer, **50,** 201. |

